# First-principles study of spin-dependent transport through graphene/BNC/graphene structure

**DOI:** 10.1186/1556-276X-8-199

**Published:** 2013-05-01

**Authors:** Tadashi Ota, Tomoya Ono

**Affiliations:** 1 Graduate School of Engineering, Osaka University, Suita, Osaka 565-0871, Japan

**Keywords:** First-principles calculation, Graphene, Hexagonal boron nitride, Spin-dependent transport property

## Abstract

First-principles study on the electronic structure and transport property of the boron nitride sheet (BNC) structure, in which a triangular graphene flake surrounded by a hexagonal boron nitride sheet, is implemented. As the graphene flake becomes small and is more isolated by the boron nitride region, the magnetic ordering of the flake increases. When the BNC structure is connected to the graphene electrodes, the spin-polarized charge-density distribution appears only at the triangular graphene flake region, and the electronic structure of the graphene electrode is not spin polarized. First-principles transport calculation reveals that the transport property of the BNC structure is spin dependent.

## Background

One of the key factors in the field of spintronics is the spin filter effect, which plays a fundamental role as the spin-polarized current source in devices such as spin-field-effect transistors and single solid-state qubits. The carbon-related nanostructures have recently been fabricated experimentally and explored theoretically to clarify magnetic ordering mainly in the zigzag edge of graphene [1-3]. These nanostructures are very attractive to the spin filter materials due to the remarkable long-spin coherence distance and high carrier mobility. On the other hand, some groups proposed the spin filter effect using quantum dots [4,5]. When the quantum dots are formed, the movements of electrons are allowed in two-dimensional gas. The movements are then restricted to zero dimension by an external field and the insulator around the quantum dots. If the small carbon flakes with a zigzag edge surrounded by an insulator have ferromagnetic ground-state electronic structures, this situation of carbon atoms resembles closely that of the quantum dots mentioned above. Okada et al. [6] studied the electronic structure of the two-dimensional triangular graphene flake surrounded by a hexagonal boron nitride sheet, which is called the BNC structure, and clarified that the zigzag edges of the graphene flake caused the magnetic ordering. Thus, the BNC structure has a large potential for the spin filter effect materials. However, in order to employ the BNC structure for the spin filter application, it is important that these BNC structures exhibit large magnetic moments and high spin-polarized transport properties when the BNC structures are connected to electrodes.

In the previous study [7], we investigated the electronic structure and transport property of the BNC structures proposed by Okada et al. [6] using first-principles calculations based on density functional theory [8] and revealed that the electron transport property of the BNC structure is spin dependent. On the other hand, there remains the other phase of the BNC structure, where the positions of boron and nitrogen atoms are exchanged. To examine the effect of the phase on the spin-polarized current through the BNC structures, the transport property of the other phase of the BNC structures is investigated in this study. Therefore, our study follows three steps: we first explore the magnetic ordering of the BNC structures under the conventional periodic boundary condition, then examine the magnetic ordering of the graphene/BNC/graphene structures, where the BNC structures are sandwiched between graphene electrodes, and finally, the spin-polarized transport property of the graphene/BNC/graphene structure is investigated.

## Methods

All calculations are performed in the framework of the density functional theory using the real-space finite-difference approach, which makes it possible to carry out the calculation with a high degree of accuracy by combining with timesaving double-grid technique and the direct minimization of the energy functional [9-11]. The valence electron-ion interaction is described by norm-conserving pseudopotentials [12] generated using the scheme proposed by Troullier and Martins [13]. Exchange and correlation effects are treated within the local spin density approximation [14].

In the calculation for electron transport properties, we employ the computational model in which the graphene/BNC/graphene structure is sandwiched between the two graphene electrodes. The scattering wave functions from the left electrode are written as follows:

(1) $$\;\Psi_{i}=\left\{\begin{array}{ll} \Psi_{i}^{\text{in}}+\underset{j}{\sum }\;r_{\text{ij}}\;\Phi_{j}^{\text{ref}} & \;\text{(in the left electrode),}\\ \phi_{i} & \;\text{(in the scattering region),}\\ \underset{j}{\sum }t_{\text{ij}}\Phi_{j}^{\text{tra}} & \;\text{(in the right electrode),} \end{array}\right)$$

where *Φ*^′^s are the bulk wave functions inside the electrode and *i* is the index of the propagating waves from the electrode. The reflection coefficients *r*, transmission coefficients *t*, and the wave function in the scattering region *ϕ* are evaluated by the overbridging boundary-matching formula under the nonperiodic condition in the *z* direction [9,15,16]. The conductance under zero temperature and zero bias is described by the Landauer-Büttiker formula [17]:

(2) $$G(E)=e^{2}/h\text{Tr}(T^{‡}T),$$

where T, *e*, and *h* are a transmission coefficient matrix, the electron charge, and Planck’s constant, respectively.

## Results and discussion

### Magnetic ordering of BNC structures

**Figure 1:**
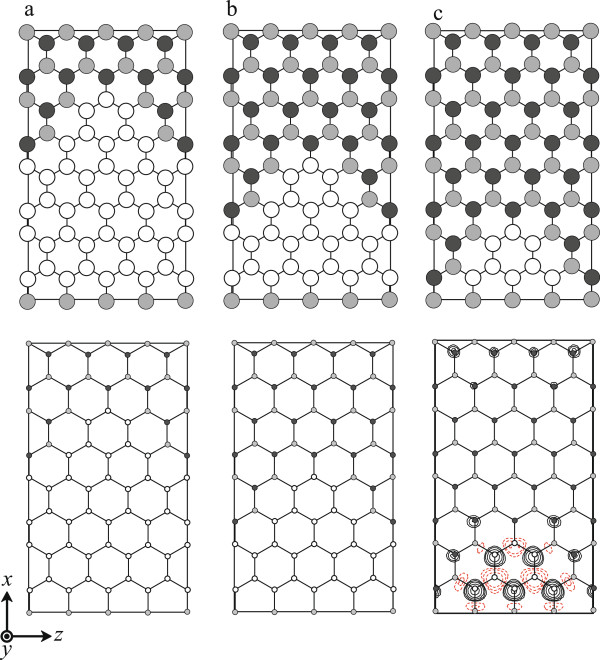
**Top view of calculated BNC structures (top) and contour plots showing difference between up-spin/down-spin charge-density distributions (bottom).** (**a**) Large, (**b**) medium, and (**c**) small graphene flake models. White, gray, and black circles represent C, B, and N atoms, respectively. Rectangle in each figure denotes the supercell. In the contour plots, positive values of spin density are indicated by solid lines and negative values by dashed lines. Each contour represents twice or half the density of the adjacent contour lines. The lowest contour represents 4.88 × 10^−2^e/bohr^3^.

In order to investigate the effect of the size of graphene flakes on the magnetic orderings, we first consider the three BNC structures under periodic boundary conditions for all directions. Figure 1 shows the computational models employed here, where 64 atoms are included in the supercell and the number of boron atoms is larger than that of nitrogen atoms. The number of *k* point used in the two-dimensional Brillouin zone integration is 16. For all the calculations in this paper, a repeating sheet model is separated by 17.0 bohr in each layer. The lattice constant is 2.67 bohr, which is obtained by the bond length of the graphene sheet. Structural optimization is performed until the remaining forces are less than 0.08 eV/bohr. Table 1 shows the calculated magnetic moments of the BNC structures. The bottom panels of Figure 1 illustrate the difference between up-spin and down-spin charge-density distributions *n*_*↑*_(*r*)−*n*_*↓*_(*r*) of the BNC structures. The BNC sheet with the smallest graphene flake is found to be the largest magnetic moment, and the spin-polarized charge-density distribution accumulates at the graphene flake region.

**Table 1:** Calculated magnetic moments of BNC structures

**Model**	**Magnetic moment (*μ***_***B***_**/cell)**
1(a)	0.00
1(b)	0.00
1(c)	1.93
2(a)	0.17
2(b)	1.09
2(c)	1.24

**Figure 2:**
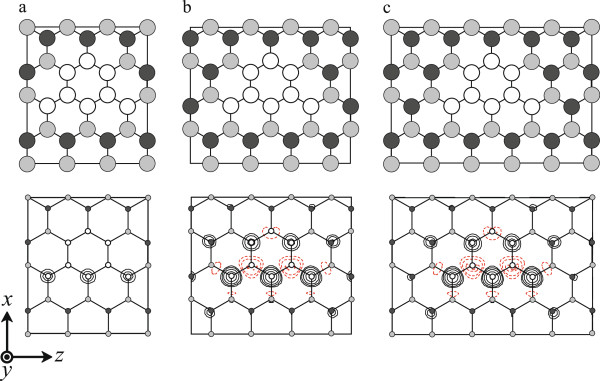
**Top view of calculated BNC structures (top) and contour plots showing difference between up-spin/down-spin charge-density distributions (bottom).** (**a**) Small, (**b**) medium, and (**c**) large distances between the smallest graphene flakes in Figure 1c. White, gray, and black circles represent C, B, and N atoms, respectively. Rectangle in each figure denotes the supercell. In the contour plots, positive values of spin density are indicated by solid lines and negative values by dashed lines. Each contour represents twice or half the density of the adjacent contour lines. The lowest contour represents 4.88 × 10^−2^e/bohr^3^.

1(a), 1(b), 1(c), 2(a), 2(b), and 2(c) correspond to the structures shown in Figures 1 and 2.

At the next step, for the purpose of investigating the effect of the distance between the graphene flakes on the magnetic moments, the other three models are investigated. Figure 2a,b,c shows the calculated atomic configurations and the difference in charge-density distribution between up-spin and down-spin electrons, *n*_*↑*_(*r*)−*n*_*↓*_(*r*). From Table 1, the BNC structure with large distance of graphene flakes shown in Figure 2c exhibits the largest magnetic moment, and the moment is strengthened when the electrons around the graphene flakes are isolated by the BN regions.

By comparing the other BNC structures investigated in a previous study [7], where the boron and nitrogen atoms are placed at opposite positions and the number of nitrogen atoms is larger than that of boron atoms, we found that the present BNC structures exhibit a similar relationship between the size of the graphene flake and magnetic moment. However, the magnetic moments are smaller than those in the previous study [7]; the energy difference of the 2 *p*_*↑*_ and 2 *p*_*↓*_ orbitals of the boron atom (1.60 eV) is smaller than that of the nitrogen atom (3.88 eV) in the local spin density approximation [14], resulting in the small splitting of the edge states.

### Magnetic ordering in graphene/BNC/graphene structure

**Figure 3:**
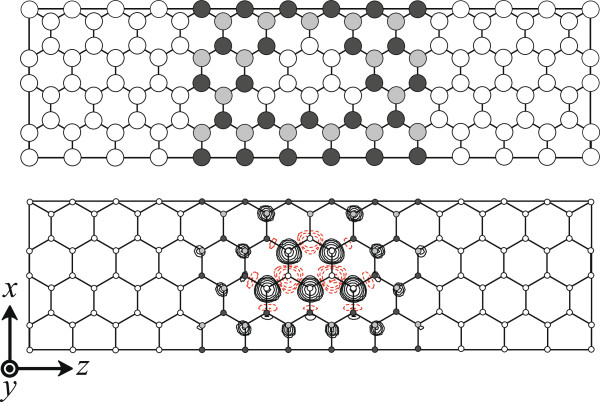
**Top view of calculated graphene/BNC/graphene structures (top) and contour plots showing difference between up-spin/down-spin charge-density distributions (bottom).** White, gray, and black circles represent C, B, and N atoms, respectively. Rectangle in each figure denotes the supercell. In the contour plots, positive values of spin density are indicated by solid lines and negative values by dashed lines. Each contour represents twice or half the density of the adjacent contour lines. The lowest contour represents 4.88 × 10^−2^e/bohr^3^.

For the investigation of the electron transport properties of the BNC structure, the electrodes have to be positioned at both sides of the BNC structure. Since the graphene structure is employed as the electrode in our study, we need to take into account whether the magnetic moments of the BNC structure are retained after the BNC structures are sandwiched between the graphene electrodes. Figure 3a shows the computational model. The integration over the Brillouin zone for the *x* direction is performed by the equidistant sampling of four *k* points. The calculated magnetic moment of the graphene/BNC/graphene structure is found to be 1.14 *μ*_*B*_. Figure 3b shows the difference between the up-spin and down-spin charge-density distributions. It should be noted that the graphene structures as the electrodes do not show the magnetic orderings, and the spin-polarized charge-density distribution accumulates at the graphene flake region.

### Transport property of graphene/BNC/graphene structure

**Figure 4:**
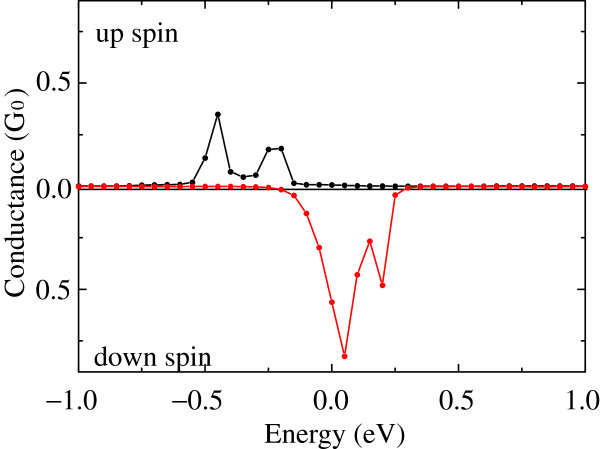
**Conductance as a function of energy of incident electrons.** Zero is chosen to be at the Fermi level.

It is important to evaluate the spin transmissions quantitatively toward the application of a spin-filter material. Based on the results in the previous subsection, the spin-polarized transport property of the graphene/BNC/graphene structure is investigated. Figure 4 shows the calculated results of the conductance and the channel transmissions. It is found that there are two peaks in the conductance spectrum, which has a similar situation with that in the previous study [7] and indicates that two bands actually contribute to the electron transport. Here, we define the parameter as follows:

(3) $$P(E_{F})=\frac{\sigma_{↑}(E_{F})-\sigma_{↓}(E_{F})}{\sigma_{↑}(E_{F})+\sigma_{↓}(E_{F})},$$

to characterize the spin polarization of the electron current, where the conductance of spin *s*(=*↑*,*↓*) is donated by *σ*_*s*_(*E*_*F*_). The spin-polarization ratio of the graphene/BNC/graphene structure is found to be approximately 0.95 at the Fermi level, which is comparable to that obtained with ferromagnetic tunnel junctions using a transition metal [18]. However, *P*(*E*_*F*_) in the present study is smaller than that in the previous study [7] due to the small energy spilt of the edge states in the band structure. In addition, all the peaks shift to the lower energy side from those in the previous study [7]. In the present model, the number of nitrogen atoms is larger, and the large electronegativity of nitrogen decreases the energy of the edge states of the graphene flake. This results in the certain conduction of the down-spin channel at the Fermi level in the present model, while the conductance at the Fermi level is negligible in the previous study [7].

## Conclusions

We have investigated the magnetic ordering and transport property of the BNC structure suspended between the graphene electrodes by first-principles calculations. The magnetic moment of the BNC structure under the conventional periodic boundary conditions increases as the size of the graphene flake becomes small and the spin-polarized charge-density distribution accumulates at the graphene flake region. It is also found that the spin-polarized charge-density distribution is preserved at the graphene flake when the BNC structure is connected to the graphene electrodes. The magnetic moment is smaller than that of the BNC structures examined in the previous study [7] because of the difference in the numbers of the boron and nitrogen atoms composing the BNC structure. The electron transport property of the graphene/BNC/graphene structure is spin-polarized. However, the spin polarization of electron current is smaller than that in the previous study [7] due to the small magnetic ordering at the BNC structure. Although there still remains much discussion to preserve spin-polarized electronic structures in the BNC structures at high temperature, these results stimulate the spin transport devices using the carbon-related materials and a bottom-up technology.

## Competing interests

The authors declare that they have no competing interests.

## Authors’ contributions

TO (T Ota) carried out preliminary calculations and drifted the manuscript. TO (T Ono) developed the computational code, implemented the calculations, and completed the manuscript. Both authors read and approved the final manuscript.
